# Prospectively isolated mesenchymal stem/stromal cells are enriched in the CD73^+^ population and exhibit efficacy after transplantation

**DOI:** 10.1038/s41598-017-05099-1

**Published:** 2017-07-06

**Authors:** Eriko Grace Suto, Yo Mabuchi, Nobuharu Suzuki, Koji Suzuki, Yusuke Ogata, Miyu Taguchi, Takeshi Muneta, Ichiro Sekiya, Chihiro Akazawa

**Affiliations:** 1 Department of Biochemistry and Biophysics, Graduate School of Health Care Sciences, Tokyo Medical and Dental University, Tokyo, 113-8510 Japan; 2 Department of Joint Surgery and Sport Medicine, Graduate School of Medicine, Tokyo Medical and Dental University, Tokyo, 113-8510 Japan; 3 Center for Stem Cell and Regenerative Medicine, Tokyo Medical and Dental University, Tokyo, 113-8510 Japan

## Abstract

Mesenchymal stem/stromal cells (MSCs), which reside in the bone marrow (BM) and various other tissues, can self-renew and differentiate into mesenchymal lineages. Many groups have harvested rat MSCs (rMSCs) from rat BM (rBM) by using a flush-out procedure and have evaluated surface marker expression after long-term culture. However, MSCs gradually differentiate during expansion and exhibit altered proliferation rates, morphological features and functions *in vitro*. Variations in MSC isolation methods may alter the effectiveness of therapeutic applications. Here, on the basis of CD29 (Itgb1) and CD54 (Icam1) expression, we prospectively isolated a population with a high colony-forming ability and multi-lineage potential from the rBM, and we demonstrated that most of these cells expressed CD73. Successful engraftment of rMSCs was achieved by using a fluorescence-conjugated anti-CD73 antibody. In humans and mice, MSCs were also purified by CD73, thus suggesting that CD73 may serve as a universal marker for prospective isolation of MSCs. Our results may facilitate investigations of MSC properties and function.

## Introduction

Mesenchymal stem/stromal cells (MSCs) were first described by Friedenstein in 1968 as cells, isolated from murine bone marrow (BM), that adhered to culture dishes and exhibited a fibroblast-like morphology^1^. MSCs reside in the BM^2^ and various other tissues such as the umbilical cord^3^, synovium^4–6^, muscle^7^, fat^8^, and dental pulp^9^. MSCs can self-renew, form colonies, and differentiate into several mesenchymal lineages including osteoblasts, chondrocytes, and adipocytes^10^. MSCs are used as a source of regenerative medicines, especially for bone and cartilage defects, heart diseases, neural diseases and GVHD, owing to their multipotency, engraftment ability and immunoregulation^11–14^. However, in most cases, transplanted cells are not engrafted for an extended period of time^15^. The presence of other contaminating cells such as fibroblasts or osteoblasts, which may potentially elicit a recipient’s immune response, may trigger MSC apoptosis. Currently, MSCs from whole BM (WBM) are obtained by maintaining adherent cells on a culture dish for several weeks, although these adherent cells are heterogeneous^16^. It is possible that eliminating other cells might improve the likelihood of successful engraftment. Moreover, MSCs gradually differentiate during expansion and exhibit altered proliferation rates, morphological features and functions from those of the parental stem cells^17^. The possibility of contamination by other cells and variations in culture isolation methods may thus result in varying degrees of effectiveness after MSC transplantation^14, 15, 18–20^. A common procedure used to isolate rat BM (rBM) cells is “flushing out” the BM^21–23^. However, MSCs residing in and attached to the endosteum are not sufficiently obtained by this method^24^. As an alternative method, we treated minced bones with collagenase and prospectively isolated rat MSCs (rMSCs) on the basis of cell surface markers. Here, cell populations isolated through the two different methods were functionally validated according to their colony-forming, differentiation and engraftment properties.

Studies in several species, including mice and humans, have identified a set of cell surface markers whose expression can be used to functionally characterize freshly isolated MSCs, such as CD271 (LNGFR), CD90 (THY1), CD106 (VCAM1), CD54 (ICAM1), and STRO-1 for human MSCs^25–27^ and CD140a (Pdgfra) and Sca-1 (Ly6e) for mouse MSCs^28, 29^. Several groups have reported that cultured rMSCs are positive for CD90 and CD54 and negative for CD44, CD45 (Ptprc), and CD34^21, 30, 31^. Nevertheless, the minimal criteria required to functionally characterize rMSCs remain unknown. The lack of uniform criteria that define MSCs across species inhibits the objective comparison of results obtained from different experiments.

Here, we identified a rat CD29^+^CD54^+^CD31^−^CD45^−^ population as rMSCs with high colony-forming and differentiation abilities, by using a collagenase treatment method. Moreover, an anti-CD73 antibody, combined with negative selection by anti-CD45 and anti-CD31 antibodies, prospectively isolated an rMSC population identical to the CD29^+^CD54^+^CD31^−^CD45^−^ population. Finally, human and mouse MSCs share the same expression pattern as rMSCs, thus suggesting that anti-CD73 may be used as a universal selection antibody to obtain purified MSCs.

## Results

### Rat colony-forming cells exhibit high CD29 expression

Commonly used methods to isolate MSCs from rBM are based on a flush-out process, which yields a heterogeneous population with a poor fibroblast colony-forming unit (CFU-F) ability. As an alternative approach to harvest MSCs effectively, bones were crushed and treated with collagenase^24, 25^. To screen the rMSC population, cell surface markers of freshly isolated rat WBM (rWBM) and cultured WBM cells were analysed by fluorescence-activated cell sorting (FACS). Freshly harvested cells expressed CD29, CD31, CD44, CD45, CD54, CD90 and CD166 (Supplementary Fig. 1a). We next isolated the positive and negative populations for each surface marker and examined their CFU-F capacity. CD29^+^ and CD54^+^ single-positive cells exhibited a high colony-forming ability, and this ability was significantly higher for CD29^+^ cells compared with CD29^−^ cells (Supplementary Fig. 1b and c).

### The CD29^+^/CD54^+^ population is enriched in rat colony-forming cells

**Figure 1:**
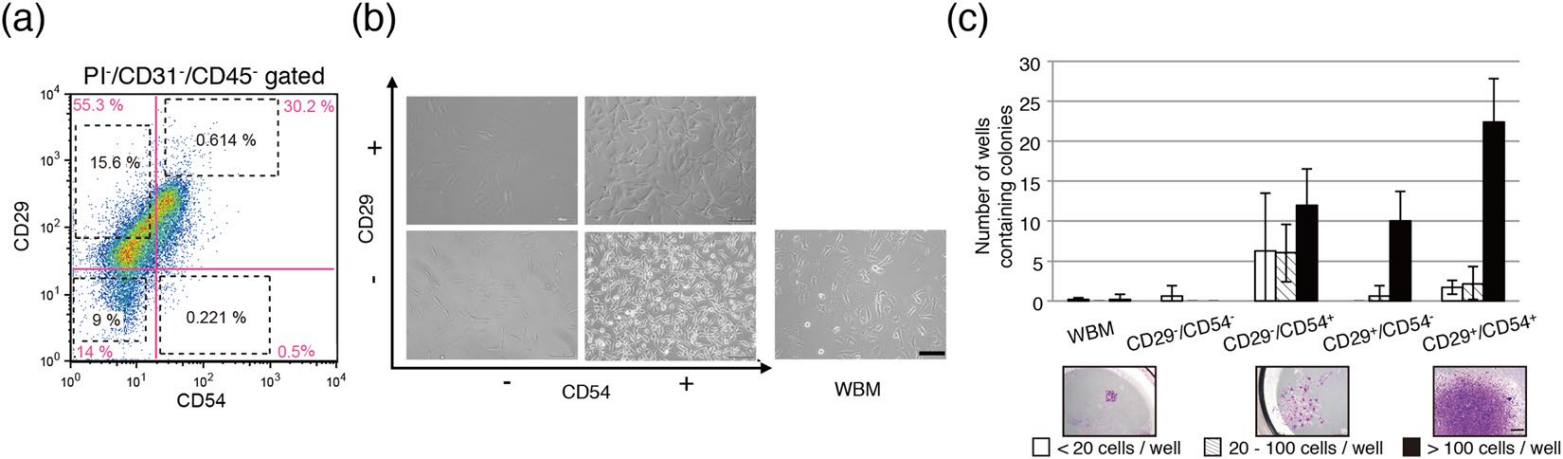
Morphological and self-renewal evaluation by clonal analysis. (**a**) Representative flow cytometric profiles of rBM cells stained for CD29 and CD54. PI^−^, CD45^−^, and CD31^−^ cells were gated. Baseline CD29 and CD54 expression is shown in pink. Sorted subsets are shown in black dotted squares. The cell number ratio is shown in the profile. (**b**) Morphological appearance of sorted sub-population cells. Scale bar, 100 µm. (**c**) Single-cell sorting assay of rBM cell sub-populations. WBM cells and four cell populations (CD29^+^/CD54^+^, CD29^+^/CD54^−^, CD29^−^/CD54^+^, and CD29^−^/CD54^−^) were sorted into 96-well plates, and the number of colonies formed was counted on day 10. Scale bar, 500 µm.

Four populations of cells were separated using anti-CD29 and anti-CD54 antibodies (CD29^+^/CD54^+^, CD29^−^/CD54^+^, CD29^+^/CD54^−^ and CD29^−^/CD54^−^ populations) and cultured on plastic dishes (Fig. 1a). Non-sorted WBM cells were used as a control. CD29^+^/CD54^+^ cells were uniformly spindle shaped, CD29^−^/CD54^+^ cells were small and spindle shaped, and CD29^+^/CD54^−^ cells were flat and blast-like shaped (Fig. 1b). CD29^−^/CD54^−^ cells rarely adhered to the plastic dishes, and those that did adhere had flat spindle-shaped morphologies but did not proliferate. To analyse colony-forming ability, single cells from each population were plated in 96-well plates (Fig. 1c). CD29^+^/CD54^+^ and CD29^+^/CD54^−^ cells formed large colonies, and CD29^−^/CD54^+^ cells formed colonies of various sizes. CD29^+^/CD54^+^ cells in particular demonstrated a high colony-forming ability. By contrast, CD29^−^/CD54^−^ cells and WBM cells rarely formed colonies. FACS was performed to analyse the cell surface marker expression of cultured cells from each subset, excluding CD29^−^/CD54^−^ cells. All populations expressed CD29, CD44, CD54, CD90 and CD166 (Supplementary Fig. 2). However, CD29 and CD54 expression was significantly lower in cultured CD29^+^/CD54^+^ cells than in other populations. Although freshly harvested WBM cells contained CD31^+^ and CD45^+^ subsets, the expression of these markers was lost during culture, thus suggesting that culturing induces significant changes in the cellular properties of rMSCs.

The CFU-F ability of the CD29^+^/CD54^+^ population was compared between cells isolated by collagenase treatment and a conventional flush-out method (Supplementary Fig. 3a). Using the flush-out method, rWBM cells were cultured on plastic dishes for 10 days. We seeded 4.4 × 10^8^ cells on average, and only 175 colonies were formed (data not shown). CD31^−^/CD45^−^ gated flushed-out cells rarely contained CD29^+^/CD54^+^ cells, and these cells had no CFU-F ability (Supplementary Fig. 3b). Moreover, CD31^+^/CD45^+^ gated cells did not form any colonies. In the flushed-out method, it is thought that the frequency of colony formation was remarkably decreased due to the large number of red blood cells. MSCs are abundant in the periosteum; therefore, it is important to use collagenase treatment to release all cells and to remove haematopoietic cells.

### CD29^+^/CD54^+^ cells can differentiate into mesenchymal lineages

The differentiation capacity of rMSC subsets was analysed *in vitro*. Accordingly, cells from each subset were grown in the indicated medium for 2 weeks and then stained with specific stains or collected for mRNA analysis of lineage-specific genes. CD29^+^/CD54^+^, CD29^+^/CD54^−^, and rWBM cells demonstrated differentiation capacities, as indicated by the presence of lipid droplets visualized by oil red O staining (Supplementary Fig. 4a). Notably, CD29^+^/CD54^−^ cells contained lipid droplets even before the induction procedure. However, unlike the other types of cells, CD29^−^/CD54^+^ cells did not form lipid droplets. Adipogenic-differentiated rWBM cells expressed high levels of adiponectin (*adipoq*), because they contained adipogenic precursor cells as well as MSCs (Supplementary Fig. 4b). CD29^+^/CD54^+^ and CD29^−^/CD54^+^ cells exhibited osteogenic differentiation ability; CD29^−^/CD54^+^ cells in particular expressed high levels of alkaline phosphatase (*alpl*). CD29^+^/CD54^+^ and WBM cells differentiated into chondrocytes, as revealed by strong safranin O staining, and expressed *aggrecan*, *sox9*, and type 2 collagen alpha 1 (*col2a1*), which are chondrogenic induction markers. Together, our results showed that CD29^+^/CD54^+^ cells uniquely differentiated into adipocytes, osteoblasts and chondrocytes. Furthermore, the other subsets may have contained mesenchymal precursor cells. As previously mentioned, CD29^+^/CD54^−^ cells exhibited lipid droplets even before the adipogenic induction, and CD29^−^/CD54^+^ cells did not differentiate into adipocytes or chondrocytes and differentiated into only osteoblasts.

### Chondrocyte-differentiated CD29^+^/CD54^+^ cells exhibit reconstruction capacity in subcutaneous tissue

**Figure 2:**
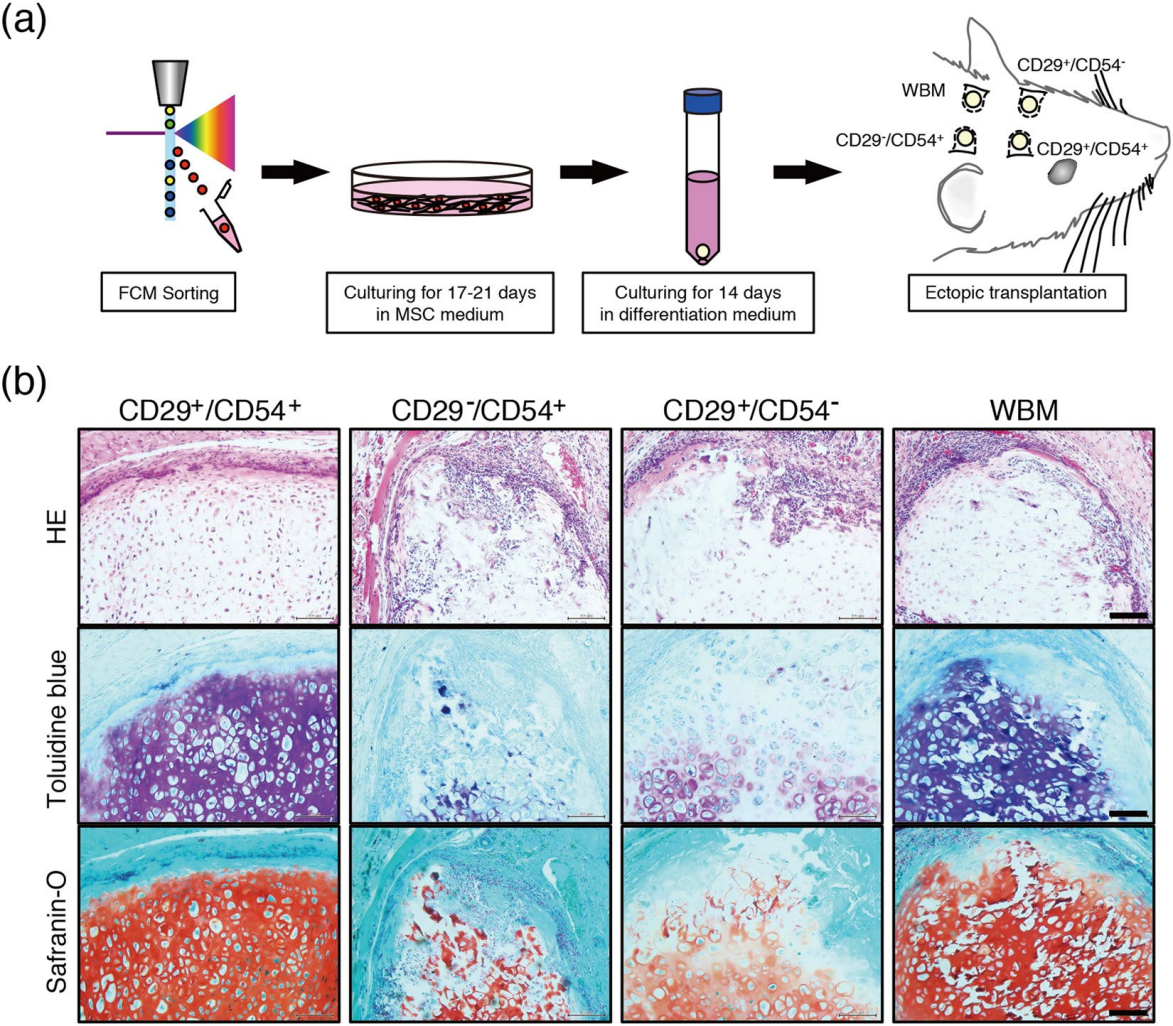
Transplantation of rMSC-derived chondrocytes into hypodermal tissue. (**a**) Scheme of cell isolation and transplantation procedures. (**b**) Representative histological sections of cell pellets transplanted after 2 weeks. The pellets were sectioned (5 µm thick) and stained with haematoxylin and eosin (HE), toluidine blue, and safranin O. Scale bar, 100 µm.

To investigate the engraftment ability *in vivo*, cell pellets cultured for 2 weeks in chondrogenic differentiation medium were subcutaneously implanted into the forehead area in rats (Fig. 2a). At 2 weeks after transplantation, the grafted pellets were excised and subjected to histological analyses. CD29^+^/CD54^+^-derived cell pellets exhibited strong staining for cartilage-like matrix, as visualized by toluidine blue staining, and chondrogenesis (safranin O staining) (Fig. 2b). By contrast, CD29^−^/CD54^+^- and CD29^+^/CD54^−^ derived pellets exhibited poor chondrogenesis and were infiltrated by erythrocytic cells (HE staining). In summary, rat CD29^+^/CD54^+^ cells display characteristics of MSCs, including self-renewal and differentiation abilities and the absence of macrophage infiltration (Table 1).

**Table 1:** Summary of the MSC potency of BM-derived sub-populations.

	CD29^+^/CD54^+^	CD29^−^/CD54^−^	CD29^−^/CD54^+^	CD29^+^/CD54^−^	WBM
CFU-Fs	++	−	+	+	−
Adipogenic induction potency	+	N.D.	−	+	+
Osteogenic induction potency	+	N.D.	++	−	±
Chondrogenic induction potency	+	N.D.	±	−	+
Non-infiltrated potency	+	N.D.	−	−	−

Self-renewal ability was evaluated as follows:++, >20 colonies;+, >10 colonies; and −, ≤1 colony in a 96-well plate (related to Fig. 1c). For differentiation abilities, the staining and mRNA expression data were evaluated as follows: ++, stained positively and demonstrated 50-fold higher expression; +, stained positively and demonstrated 2-fold higher expression; ±, stained positively and tended to demonstrate higher expression of the differentiation marker than the subset in which expression was lowest; and −, not stained and demonstrated the lowest mRNA expression level (related to Supplementary Fig. 4). Macrophage infiltration was evaluated as follows: +, macrophages not seen in the transplanted area; and −, macrophages infiltrated into the graft (related to Fig. 2).

### rMSCs express high levels of CD73

**Figure 3:**
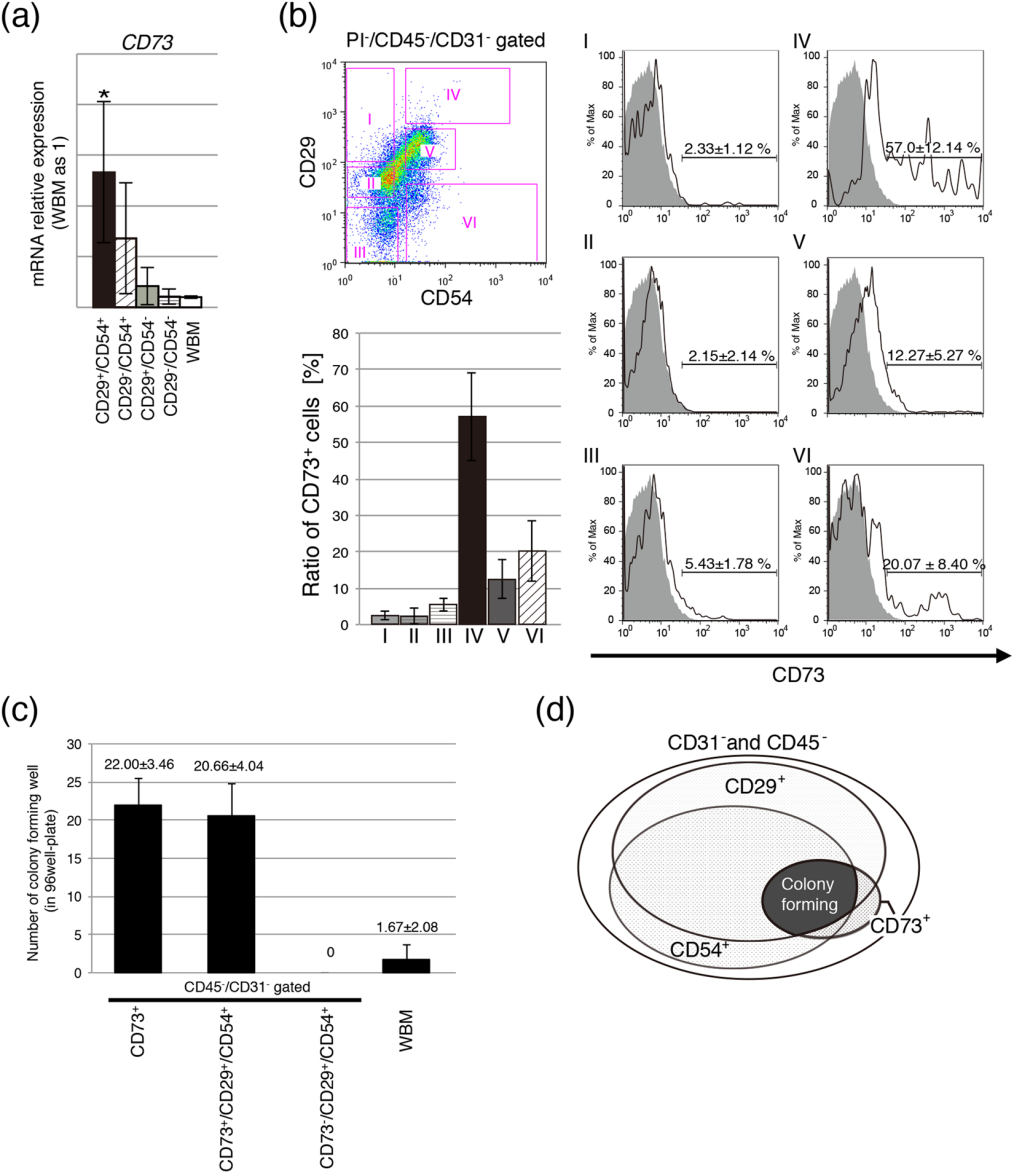
CD73 marker identifies the CD29^+^CD54^+^ population. (**a**) Relative mRNA expression of CD73 markers in freshly isolated cells. Gene expression was normalized to that of Hprt (n = 3; *P < 0.05). (**b**) CD73^+^ cells in the various sub-populations gated using anti-CD29 and anti-CD54 antibodies. (**c**) Colony-forming assay of single cells seeded into 96 well plates. (**d**) Venn diagram of CD29, CD54 and CD73 expression in non-haematopoietic rBM cells.

Next, we tested whether a single antibody might be used in prospective isolation of rMSCs. Each subset of cells sorted on the basis of anti-CD29 and anti-CD54 antibodies was subjected to mRNA analysis for CD73, which is also known as an rMSC marker. High *CD73* mRNA expression was detected in the CD29^+^/CD54^+^ subset of cells (Fig. 3a). Triple-staining for CD73, CD29 and CD54 protein expression revealed that CD73^+^ cells were enriched in the CD29^high+^/CD54^+^ subset (population IV) (Fig. 3b). To analyse the colony formation ability, single sorted cells were plated in 96-well plates. CD73^+^ cells in non-haematopoietic populations (CD73^+^/CD31^−^/CD45^−^ and CD73^+^/CD29^+^/CD54^+^/CD31^−^/CD45^−^) exhibited a high colony-forming ability, whereas CD73^−^ cells formed no colonies (Fig. 3c). On the basis of these data, a Venn diagram was constructed (Fig. 3d). These results suggested that rMSCs expressed CD73 and that an antibody against CD73 can be used to prospectively isolate and enrich rMSCs.

**Figure 4:**
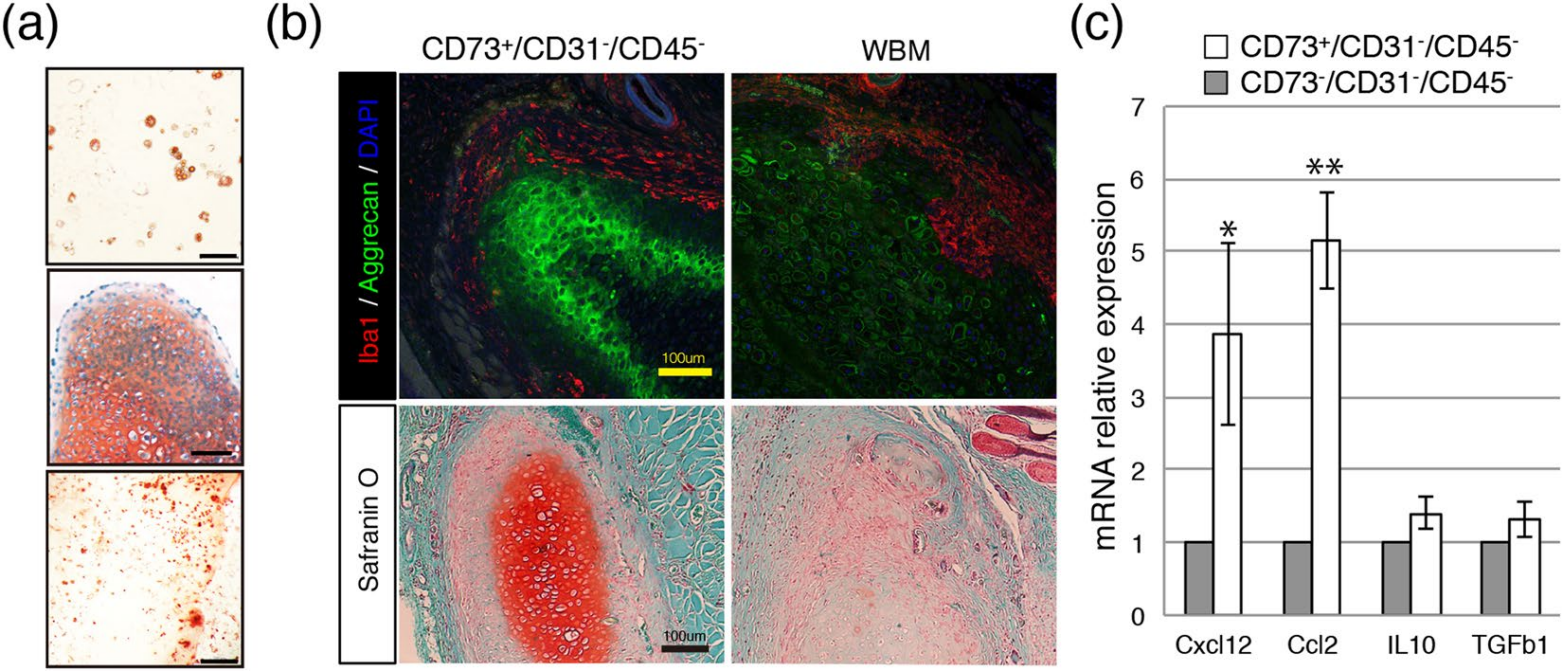
Differentiation ability and lack of infiltration of CD73^+^ rMSCs. (**a**) Capacity of CD73-MSCs to differentiate into adipocytes, chondrocytes, and osteoblasts. Scale bar, 100 µm. (**b**) Double fluorescence staining to label the chondrogenic marker aggrecan (green) and the macrophage marker Iba1 (red) (top). Scale bar, 100 µm. Histological sections of cell pellets transplanted after 2 weeks. The pellets stained with safranin O (low). Scale bar, 100 µm. (**c**) Quantitative analysis of the mRNA expression of *Cxcl12*, *Ccl2*, *Il10*, and *Tgf b1* in freshly isolated MSCs (CD73^+^ and CD73^−^ cells). The mRNA expression of each gene was normalized to that of Hprt. The gene expression in CD73^−^CD45^−^CD31^−^ cells was set as 1.0.

We next investigated the ability of CD73^+^ cells to differentiate into mesenchymal lineages *in vitro* and found that these cells exhibited pronounced differentiation into adipocytes, osteoblasts, and chondrocytes (Fig. 4a). To determine efficacy after transplantation, induced CD73^+^ cell pellets were ectopically transplanted *in vivo*, and chondrogenic differentiation and a lack of infiltration were observed (Fig. 4b). Thus, in terms of therapeutic application, prospective isolation with a single CD73 antibody would increase the likelihood of a successful engraftment. Histochemical analyses revealed that differentiated pellets derived from the prospective isolation blocked the infiltration of inflammatory cells such as Iba1-positive macrophages. Furthermore, CD73^+^ cells expressed key MSC molecules in the haematopoietic niche and cellular therapy, such as *CXCL12* ^32^ and *CCL2* ^33^ (Fig. 4c). These data suggested that CD73^+^ cells are transplantable MSCs that can be isolated from rBM.

### CD73 is a universal marker for the prospective isolation of MSCs

**Figure 5:**
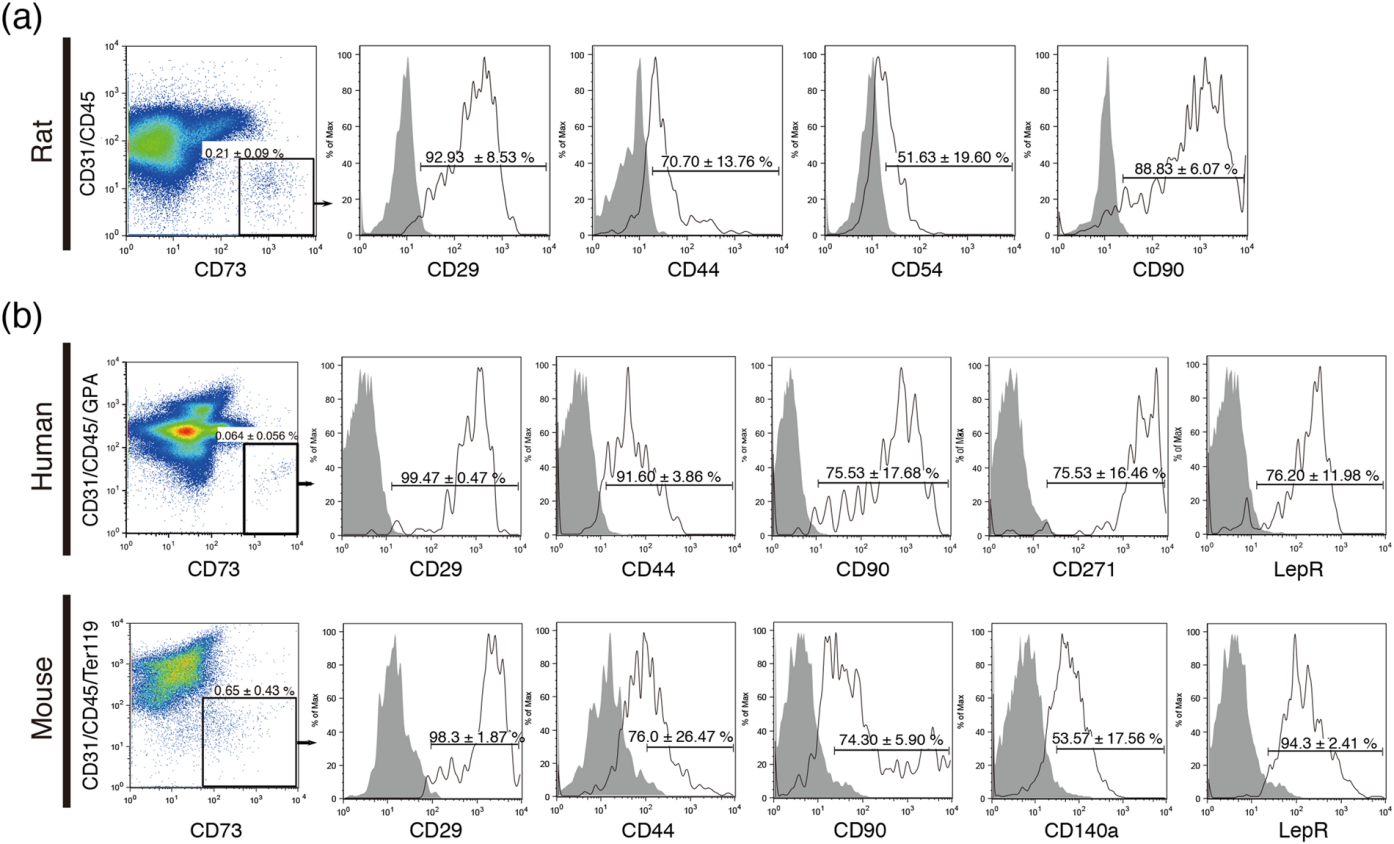
CD73 is commonly available for the prospective isolation of MSCs. (**a**) Representative results of flow cytometric analysis for cell surface markers using freshly isolated BMMNCs from rats. The CD73^+^ population is positive for previously described MSC markers (CD29, CD44, CD54, and CD90). (**b**) Flow cytometric analysis in the CD73^+^ population for cell surface markers from human and mouse BM (CD29, CD44, CD90, CD140a, CD271, and leptin receptor).

Although surface markers of human and mouse MSCs have been well characterized, cell populations prospectively isolated by using human and mouse anti-CD73 antibodies have not been comprehensively studied. Flow cytometric analysis of fresh rBM cells revealed that the CD73^+^ population expressed CD29, CD44, CD54, and CD90 (Fig. 5a). Human BM mononuclear cells or mouse BM cells isolated by collagenase methods were stained with anti-CD73, anti-CD31, anti-CD45 and human GPA (glycophorin A) or mouse Ter119 antibodies to prospectively isolate non-haematopoietic CD73^+^ populations. As shown in Fig. 5b, CD73^+^ cells expressed most of the previously described MSC markers, such as CD29, CD44, CD90 and CD271 (human) or CD140a (mouse). Moreover, both human and mouse CD73^+^ cells expressed high levels of the leptin receptor (Fig. 5b). These results suggested that CD73 may be a candidate marker for standardizing data from human, mouse and rat MSCs.

## Discussion

MSCs have been isolated from various species including mice, rabbits, dogs, and humans^34, 35^. Although the variations among MSC populations derived from different species are becoming more evident, it is unclear whether they are functionally and intrinsically equivalent. Therefore, efforts to test robust markers representing properties of MSCs are necessary, and data derived from one species may require careful validation. Here, we prospectively isolated rMSCs from collagenase-treated rBM by using CD29^+^/CD54^+^/CD73^+^/CD31^−^/CD45^−^ as selection markers. The cell population isolated by CD29^+^/CD54^+^ selection was identical to that isolated by single CD73^+^ selection. The colony-forming efficiency of the population isolated by single CD73^+^ selection was similar to that of human MSCs (LNGFR^+^/THY-1^+^)^25^ and mouse MSCs (PDGFRα^+^/Sca-1^+^)^24^. Flow cytometric analysis of fresh BM cells revealed that the CD73^+^ population uniformly expressed known MSC markers (CD29, CD44, and CD90) (Fig. 5). Although CD90 has been reported as an rMSC marker^36^, colony-forming cells were not enriched on the basis of anti-CD90 antibody selection. Thus, surface marker expression should be carefully evaluated to isolate and compare MSCs among different species.

Previous reports, such as that by Harting *et al*.^37^, and our present results showed that culturing induces cell surface marker changes (Supplementary Fig. 2). Because the cells in our study were freshly purified by using selection markers before plating, it is therefore the MSCs that exhibited altered surface marker expression over time. Therefore, it must be noted that the cell properties of MSCs would differ depending on whether they were purified before or after culturing. Some groups have reported that CD29 and CD54 in cultured MSCs function in migration and engraftment^38, 39^. We found that cultured cells expressed both CD29 and CD54 even if they were initially negative for these markers when they were freshly sorted. Thus, to observe the *bona fide* behaviour of MSCs *in vivo*, careful comparison between freshly isolated MSCs and those cultured through passages is necessary. In the case of prospectively isolated CD29^+^/CD54^+^ cells, ectopic engraftments of the pellet after transplantation formed mature chondrocyte-like structures *in vivo* and were not infiltrated by macrophages after four weeks (Figs 2 and 4). The structure of pellets was deformed for the engraftments that originated from populations such as CD29^−^/CD54^+^, CD29^+^/CD54^−^ and CD29^−^/CD54^−^ cells, which may contain chemoattracting immune cells^40, 41^ such as haematopoietic cells, osteoblasts, or adipocytes. Prospective isolation on the basis of MSC markers appears to be advantageous for a stable engraftment that is not infiltrated by macrophages.

Some groups have reported that soluble CD54 (sCD54) may inhibit macrophage activity^42, 43^ and that leptin promotes CD54 expression and subsequently enhances the level of sCD54^44^. Leptin is highly induced by adipose cells. In our study, adipose precursor cells were found in the CD29^+^/CD54^−^ population and expressed CD54 after culturing. As shown in Fig. 2b, CD29^+^/CD54^−^ derived pellets were not infiltrated, as compared with the CD29^−^/CD54^+^ population, which may contain osteoblast cells. CD29, which is also known as Integrin β1, is highly expressed in not only rMSCs but also in macrophages and plays an important role in cell migration^45^. It is also the principal receptor for binding extracellular matrix (ECM) components, such as laminins, fibronectin, and collagens, and is involved in cell attachment^46^. ECM components probably surrounded the CD29^+^-derived cell pellet, and macrophages migrated to attach to them without infiltrating. Co-expression of CD54, which is known as an integrin ligand, may support cell pellet survival. Therefore, ECM proteins may be present between CD29^+^/CD54^+^-derived cell pellets and macrophages and thus inhibit macrophage infiltration into transplanted cell pellets. Our findings suggest a mechanism by which macrophage infiltration can be inhibited to raise stable engraftments.

It has been reported that CD73 produces adenosine and functions in immune tolerance^47, 48^. We constructed a fluorescence-conjugated anti-CD73 antibody and used it to isolate cells with a high colony-forming ability that did not become infiltrated. MSCs have been reported to be immune tolerant^49^, a result consistent with our findings regarding CD29^+^/CD54^+^ cells. Therefore, rMSCs were highly enriched in the CD73^+^ population *in vivo*. Although our *in vivo* experiments involved allogeneic transplantation (an inbred strain), grafted CD73^+^ cell pellets were not rejected. This result was also consistent with MSC immune tolerance. Further work is required to understand the roles of CD73^+^ cells in the immune response. rMSCs can be simply isolated from bones by using only an anti-CD73 antibody. Almost half of the CD73^+^ cells expressed both CD29^+^ and CD54^+^; however, the interactions between these cell surface markers remain unknown.

MSCs expanded over a long period of time have been clinically used as therapeutic agents and have resulted in variable outcomes. Many groups harvest rMSCs by using a flush-out procedure and evaluate surface marker expression after long-term culturing. Sorting harvested BM cell populations after collagenase treatment with specific markers may facilitate the isolation of sufficient and good-quality rMSCs, because the flush-out methods do not efficiently detach the MSCs from the endosteum of bones. Previous reports have shown that MSCs are responsible for supporting haematopoiesis in the BM^50^. Leptin receptor-positive cells create a haematopoiesis microenvironment for HSCs^51, 52^. Because human and mouse CD73^+^ cells express the leptin receptor (Fig. 5b), identifying CD73^+^ cells as an indicator of MSCs may advance understanding of their relationship with HSCs and their use in cell therapy for haematopoietic diseases.

MSC therapy has entered the clinic in a wide variety of applications related to tissue repair and immunoregulation. According to the National Institutes of Health’s clinical trial registry, more than 700 clinical trials from Phase 1 to Phase 4 involving MSC therapy are currently being performed worldwide (http://clinicaltrials.gov). Our protocol, not only for rats but also for mice and humans, shows that CD73^+^ MSCs exhibit good self-renewal and differentiation abilities and are able to protect against infiltration. In conclusion, we demonstrated the feasibility of enriching for high-quality MSCs by using CD73^+^ as a marker for prospective isolation.

## Methods

### Animals

Male Lewis rats (9–10 weeks of age) and C57BL/6J mice (8 weeks of age) were purchased from Japan Charles River. All experimental protocols were approved by the animal committee of Tokyo Medical and Dental University, Japan (protocol number: 0170282C). All methods were conducted in strict accordance with the approved guidelines of the institutional animal care committee.

### BM cell isolation and expression of surface markers

For the flush-out method, rat femora without both ends were flushed out with Hank’s Balanced Salt Solution (HBSS) containing 2% foetal bovine serum (FBS) by using an 18-G needle syringe, as previously described^21^. For collagenase treatments, rat femora, tibias and ilia were chopped and minced in HBSS containing 2% FBS after 2 mg/mL collagenase (Wako) treatment for 1 hr at 37 °C. Mouse BM cells were isolated from femora, tibias and ilia by collagenase treatment. Human BM cells were purchased as Bone Marrow Mononuclear Cells (LONZA). The cells were diluted in HBSS containing 2% FBS, 10 mM HEPES, and 1% penicillin/streptomycin (GIBCO) and stained with the following mouse antibodies for rBM: APC-conjugated anti-CD90.1, FITC-conjugated anti-CD54, PE-conjugated anti-CD31, PE-Cy7-conjugated anti-CD45, PE-conjugated anti-CD29 (all from BD Pharmingen), PE-conjugated anti-CD44H (eBioscience), goat anti-CD166 (R&D), and Alexa Fluor 647-conjugated donkey anti-goat IgG (Abcam). Propidium iodide (PI) fluorescence was used to gate dead cells. FITC-conjugated anti-CD54, PE-conjugated anti-CD29, biotin-conjugated mouse anti-rat CD31, and biotin-conjugated mouse anti-rat CD45 antibodies and PE-Cy7-conjugated streptavidin (all from BD) were used for multiple colour staining. An Allophycocyanin Labeling Kit-NH2 (DOJINDO) was used to label the anti-CD73 antibody with fluorescence. Flow cytometry and sorting were performed on FACS Verse and FACS Aria II instruments (BD).

### Cell culture

Sorted cells were plated on non-coated dishes and incubated at 37 °C in 5% CO_2_. The MSC medium consisted of Dulbecco’s Modified Eagle’s Medium-GlutaMAX (Life Technologies) containing 20% FBS (HyClone), 20 ng/mL basic fibroblast growth factor (Repro CELL), and 1% penicillin/streptomycin. The medium was changed twice per week.

### Multi-lineage differentiation of rBM cells

For adipogenic differentiation, 1.0 × 10^4^ cells at passage 3 were plated in a 24-well plate in adipogenic induction medium (LONZA) and incubated at 37 °C in 5% CO_2_. For osteogenic differentiation, 0.7 × 10^4^ cells at passage 3 were plated in a 24-well plate in osteogenic induction medium (LONZA) and incubated at 37 °C in 5% CO_2_. Both adipogenic- and osteogenic-induced cells were cultured for 2 weeks. For chondrogenic differentiation, 5.0 × 10^5^ cells at passage 3 were placed in a 15-mL polypropylene tube (Nunc) and centrifuged at 200 × g for 4 min. The cells were cultured in chondrogenic induction medium (LONZA) containing 500 ng/mL bone morphogenetic protein-6 and 10 ng/mL transforming growth factor-b3 (TONBO) and incubated at 37 °C in 5% CO_2_ for 2 weeks. All media were changed twice per week. Histological staining was performed with oil red O (Muto Pure Chemicals) for adipogenic-induced cells, alizarin red (Millipore) for osteogenic-induced cells, and safranin O/fast green (Muto Pure Chemicals) for chondrogenic-induced cells.

### Quantitative RT-PCR

Quantitative RT-PCR was performed using Super Script III Reverse Transcriptase (Thermo Fisher Scientific) and Fast SYBR Green Master Mix (Thermo Fisher Scientific). The primers are described in Table 2 (MSC related genes) and Supplementary Table (mesenchymal lineage-specific genes).

**Table 2:** Primer sequences for quantitative RT-PCR.

		Sequence	Product length (bp)
*hprt*	Forward	5′-CCAGTCAACGGGGGACATAAA-3′	142
	Reverse	5′-GGGGCTGTACTGCTTGACCAA-3′	
*CD73*	Forward	5′-ATGCTGGCGATCAGTACCA-3′	197
	Reverse	5′-CCGGGCCTTAATATTTGCA-3′	
*CXCL12*	Forward	5′-GTTTGCTTTGGAGCTTCTCG-3′	99
	Reverse	5′-GCTCTGGTGGAAGGTTGCTA-3′	
*Ccl2*	Forward	5′-CTACTCATTCACTGGCAAGA-3′	110
	Reverse	5′-TCTTGAGCTTGGTGACAAAT-3′	
*IL*-*10*	Forward	5′-TCTACAAGGCCATGAATGAG-3′	85
	Reverse	5′-CGGGTGGTTCAATTTTTCAT-3′	
*TGF*-*b1*	Forward	5′-CTACTGCTTCAGCTCCACAG-3′	320
	Reverse	5′-TGCACTTGCAGGAGCGCAC-3′	

### Transplantation and histological examination

Cells were cultured in chondrogenic differentiation medium for 2 weeks and formed a pellet. Under isoflurane anaesthesia and after the removal of hair, the rat forehead was exposed, and chondrogenic-differentiated cell pellets were implanted subcutaneously. After 2 weeks, the grafted areas were dissected and embedded in paraffin. The embedded block was sliced into 5 µm thick sections. Haematoxylin and eosin staining (Wako), safranin O and toluidine blue staining (Wako), and immunostaining (anti-aggrecan antibody, Hybridoma; anti-Iba-1 antibody, Wako) were performed. Confocal microscopy was used to visualize the immunohistochemical results (Zeiss).

### Statistical analysis

Quantitative data are presented as the means ± the standard error of the mean (SEM) from at least 3 representative experiments. For statistical analysis, the data were evaluated with Student’s t-test. In all cases, p-values of <0.05 were considered significant.

## Electronic supplementary material

Supplementary figures and table
