# Supplementary material for: Prospectively isolated mesenchymal stem/stromal cells are enriched in the CD73+ population and exhibit efficacy after transplantation
Source: Sci Rep. 2017 Jul 6;7:4838. doi: 10.1038/s41598-017-05099-1 (PMC5500568; doi:10.1038/s41598-017-05099-1)
Supplement: Supplementary file 1 — Supplementary figures and table [file 41598_2017_5099_MOESM1_ESM.pdf]

Supplemental figures and table for

Prospectively isolated mesenchymal stem/stromal cells are enriched in the CD73$^+$ population and exhibit efficacy after transplantation

Authors

Eriko Grace Suto, Yo Mabuchi, Nobuharu Suzuki, Koji Suzuki, Yusuke Ogata, Miyu Taguchi, Takeshi Muneta, Ichiro Sekiya & Chihiro Akazawa

Supplementary Fig.1: Cell surface marker analysis of rat BM cells

Supplementary Fig.2: Surface marker analysis of cultured sub-population of rat BM cells

Supplementary Fig.3: Comparison of the potency of MSCs isolated by collagenase treatment or the flush-out method

Supplementary Fig.4: Differentiation potency of cultured BM cells sub-population into mesenchymal lineages

Supplementary table: Primers sequences for RT-PCR

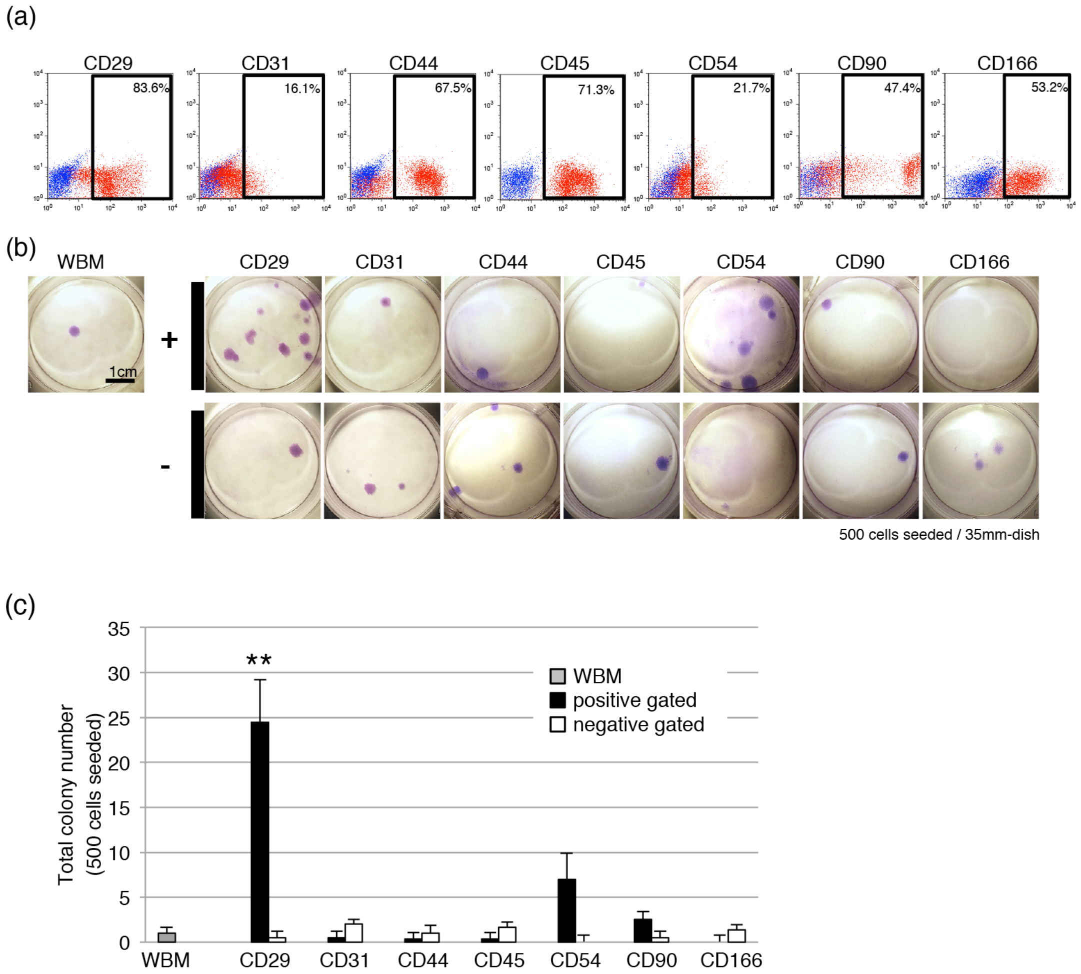

Supplementary Fig.1| Cell surface marker analysis of rat BM cells.

Analysis of cell surface marker expression of freshly harvested rat BM cells. (a) Representative cell surface expression of CD29, CD31, CD44, CD45, CD54, CD90, and CD166 in freshly isolated BM cells determined by FACS. Stained BM cells are shown as red dots and negative control cells are shown as blue dots. (b) Representative images of CFU-Fs stained with crystal violet (one of three individual experiments). Freshly harvested cells were stained with antibodies for CD29, CD31, CD44, CD45, CD54, CD90, and CD166. Positive (+) and negative (-) cells were sorted and cultured for 10 days on 35mm-dishes. (c) The number of CFU-Fs per dish was counted (n=3; ** $P<0.01$).

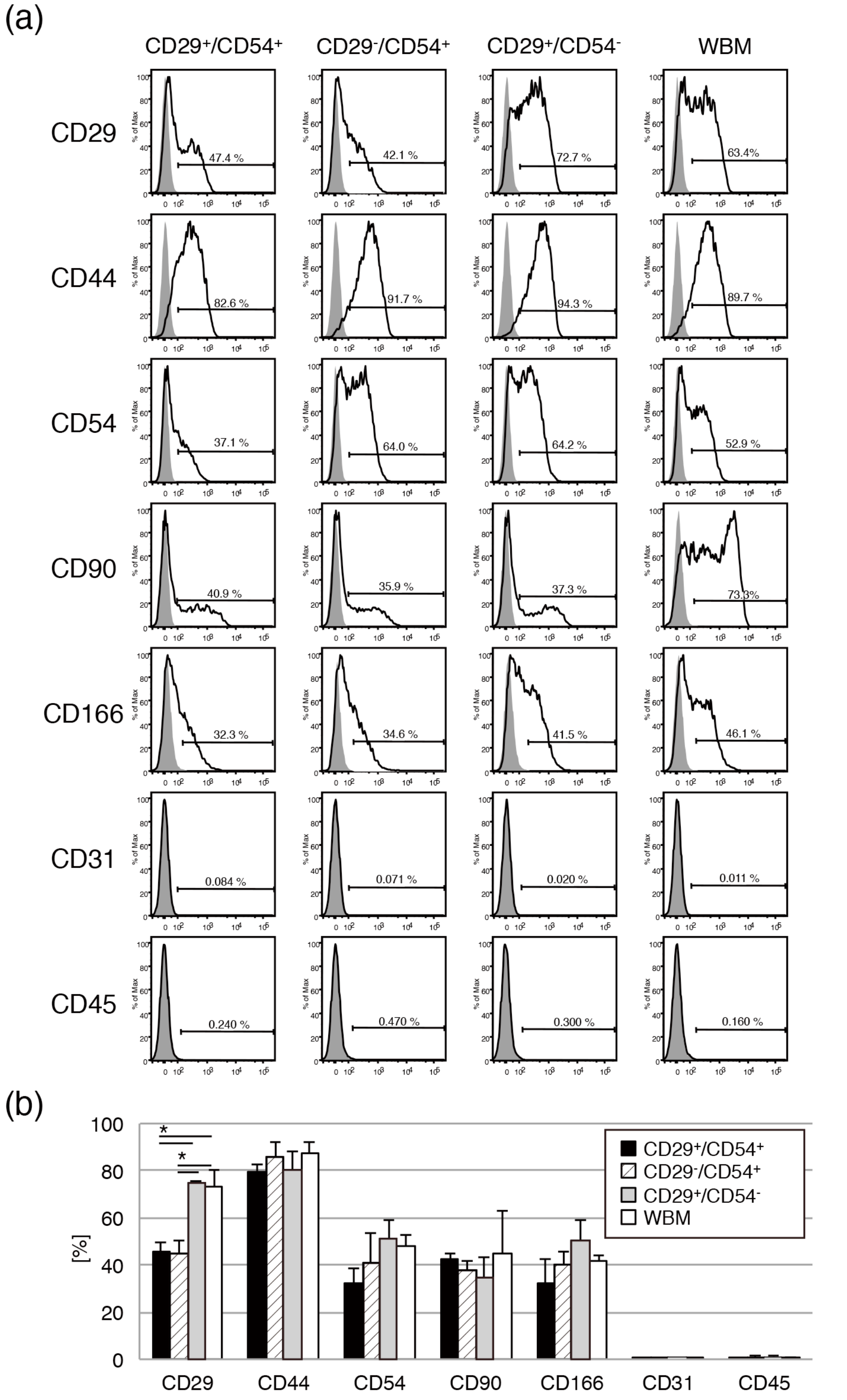

Supplementary Fig.2| Surface marker analysis of cultured sub-populations of rat BM cells.

Rat BM cells ($CD29^+/CD54^+$, $CD29^-/CD54^+$, $CD29^+/CD54^-$ and WBM) were cultured for 3 weeks. After harvesting with cell dissociation buffer, cultured cells were labeled with antibodies against mesenchymal cell markers (CD29, CD44, CD54, CD90, and CD166), endothelial and hematopoietic cell markers (CD31 and CD45). (a) Representative flow cytometric profiles showing the percentage of cells expressing the antigen (line) vs. a matched isotype control (gray shading). (b) Summary of the surface marker expression of cultured sub-populations (n=3; * $P<0.05$).

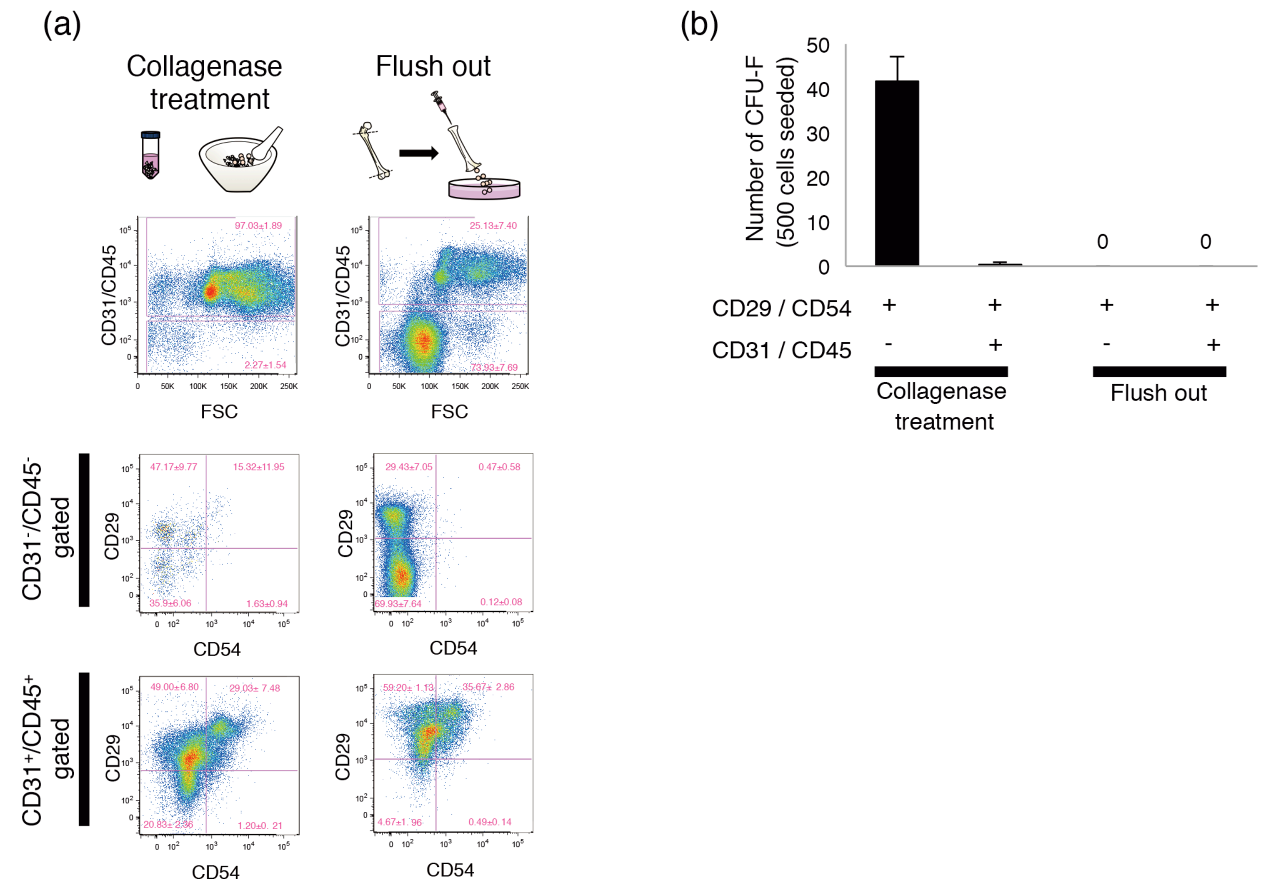

Supplementary Fig.3| Comparison of the potency of MSCs isolated by collagenase treatment or the flush-out method.

Rat femurs, tibias, and ilia were crushed and treated with collagenase or subjected to the flush-out method. (a) Harvested cells were sorted as described. CD29 and CD54 expression from $CD31^-/CD45^-$ or $CD31^+/CD45^+$ population were analyzed. (b) Each subset (500 cells) was seeded, and the number of CFU-Fs was counted after 10 days (n=3).

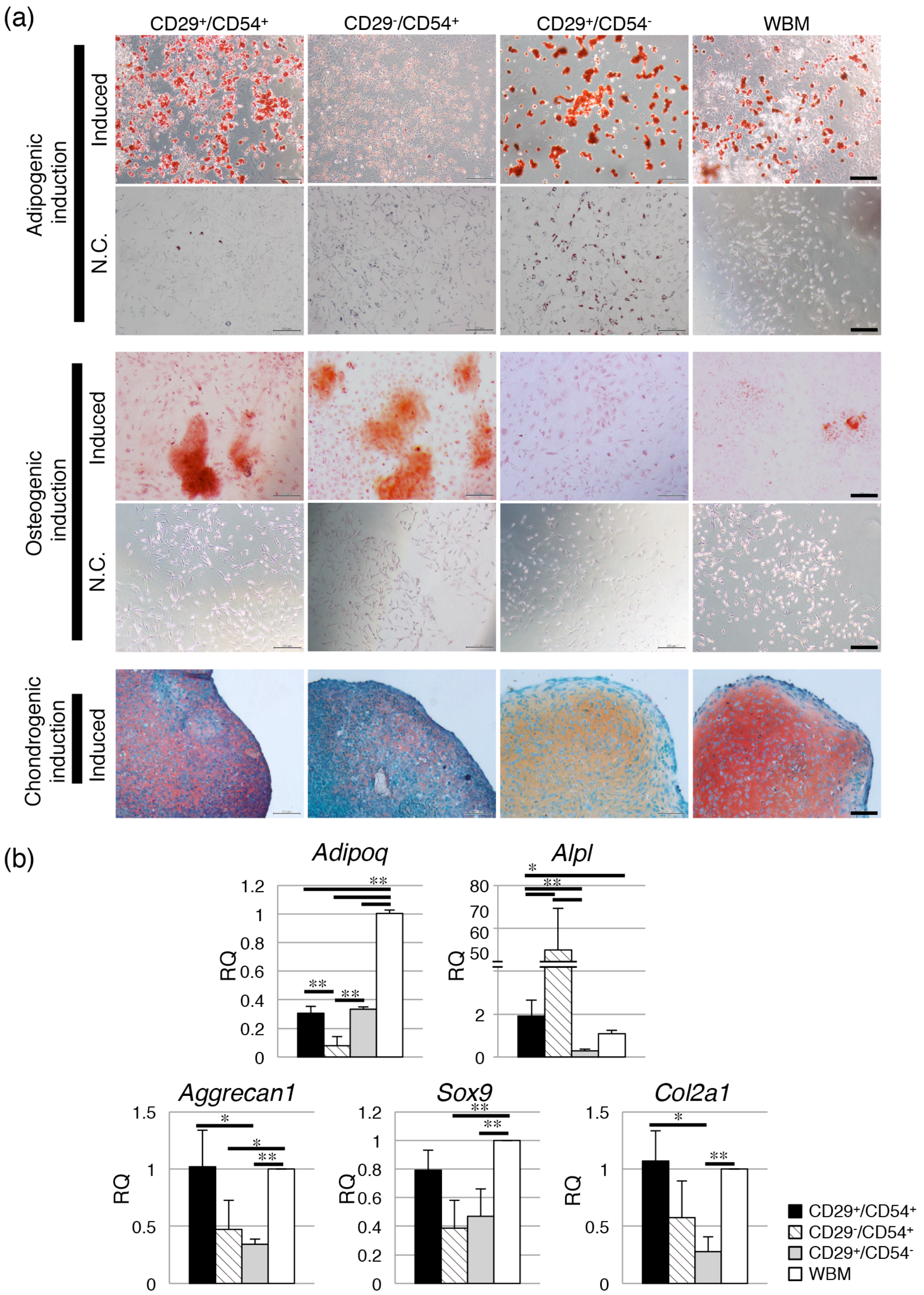

Supplementary Fig.4| Differentiation potency of cultured BM cell sub-populations into mesenchymal lineages.

Cultured cells at passage 2 were differentiated into adipocytes, osteoblasts, or chondrocytes in defined medium. (a) Representative photomicrographs of cells differentiated for 2 weeks (induced) or undifferentiated negative control (N.C.). Scale bars; 200 µm for adipogenic- and osteogenic-differentiated cells, and 150 µm for chondrogenic-differentiated cells. (b) Expression ratio of *adipoq* mRNA in adipogenic-induced cells, *alp* mRNA in osteogenic-induced cells, and *aggrecan*, *sox9*, and *col2a1* mRNA in chondrogenic-induced cells. Gene expression was compared with that in differentiated WBM cells. Expression levels were normalized to that of the reference gene *Hprt* (n=3; * $P<0.05$; ** $P<0.01$).

Supplementary table| Primers sequences for RT-PCR

		Sequence	Product length (bp)
adipoq	Forward	5'- TGTTCCTCTTAATCCTGCCCA -3'	95
	Reverse	5'- CCAACCTGCACAAGTTTCCTT -3'	
alpl	Forward	5'- CAGGATTGACCACGGGCACC -3'	327
	Reverse	5'- GCCTGGTAGTTGTTGTGAGC -3'	
aggrecan	Forward	5'- GCCATCATCGCCACCCCTGAGCAA -3'	350
	Reverse	5'- ATGTCCATACCGCCCTGCCAGGCA -3'	
sox9	Forward	5'- TCTCCTAACGCCATCTTCAAGGCG -3'	244
	Reverse	5'- TGCTCAGCTCACCGATGTCCAC -3'	
col2a1	Forward	5'- TTGAGACAGCATGACGTCGAG -3'	360
	Reverse	5'- AGCCAGGTTGCCGTCGCCGTA -3'	
